# E4orf1: A protein for enhancing glucose uptake despite impaired proximal insulin signaling

**DOI:** 10.1371/journal.pone.0208427

**Published:** 2018-12-06

**Authors:** Anuradha A. Shastri, Vijay Hegde, Swetha Peddibhotla, Zahra Feizy, Nikhil V. Dhurandhar

**Affiliations:** Department of Nutritional Sciences, Texas Tech University, Lubbock, Texas, United States of America; Tohoku University, JAPAN

## Abstract

**Background:**

Type 2 diabetes is often linked with impaired proximal insulin signaling. Hence, a therapeutic agent that enhances cellular glucose uptake without requiring proximal insulin signaling would be desirable for improving glycemic control. The E4orf1 peptide (E4) derived from human adenovirus 36 (Ad36) promotes cellular glucose uptake in vitro and in vivo, independent of insulin. E4 bypasses a part of insulin signaling to upregulate cellular glucose uptake. We tested the hypothesis that E4 requires the distal but not proximal insulin signaling to enhance cellular glucose disposal.

**Methods:**

3T3-L1 preadipocytes inducibly expressing E4 or a null vector (NV) were treated with inhibitor of insulin receptor (S961), inhibitor of insulin like growth factor-1receptor (IGF-1R) (Picropodophyllin, PPP), PPP+S961, or phosphatidyl inositol-3 kinase (PI3K) inhibitor (Wortmannin, WM). We used PPP and S961 to block the proximal insulin signaling, or WM to block the distal insulin signaling. Cells were exposed to 0 or 100nM insulin.

**Results:**

As expected, when the proximal or distal insulin signaling was blocked in NV cells, insulin could not enhance pAKT protein abundance, Glut4 translocation, or glucose uptake. Whereas, E4 cells significantly increased pAKT abundance, Glut4 translocation and glucose uptake independent of the presence of insulin or proximal insulin signaling. Enhanced glucose disposal in E4 cells was completely abrogated when the distal insulin signaling was blocked.

**Conclusions:**

E4 bypasses the proximal insulin signaling but uses the distal insulin signaling to activate pAkt and in turn Glut4 translocation to improve cellular glucose uptake. E4 offers a promising template to improve glycemic control when the proximal insulin signaling is impaired.

## Introduction

Insulin resistance is characterized by elevated circulating insulin [1] and glucose due to impaired functioning of the insulin receptors [2, 3]. Excessive insulin production overtime leads to decrease in insulin release from pancreas and β cell exhaustion. Diabetes is associated with progression to decreased insulin production with/without impaired insulin receptor function, leading to higher blood glucose levels [3].

The normal insulin signaling leading to glucose uptake involves binding of insulin to its receptor, autophosphorylating it, which in turn autophosphorylates the insulin receptor substrates (IRS) such as IRS1 and IRS2 on their tyrosine residue. Phosphorylation of the receptors triggers the activation of PI3K, which phosphorylates Akt leading to glucose transporter 4 (Glut4) translocation to the cell membrane for uptake of glucose. The insulin receptors (IR) and IRS constitute the proximal insulin signaling while PI3K, Akt and Glut4 are considered part of the distal insulin signaling.

Insulin resistance linked to obesity or type 2 diabetes is associated with decreased cellular glucose uptake in the adipose and muscle tissues and increased glucose output from the liver [4, 5], which may be caused in part by impaired proximal insulin signaling [2, 3]. Diabetes is commonly treated with medications such as insulin secretagogues and sensitizers (e.g. biguanides and thiazolidinediones (TZD)) which engage the proximal insulin signaling [6–8]. Also, treatment with anti-diabetic drugs is most effective when combined with weight loss and dietary modifications, but long term compliance with these lifestyle modifications is difficult [9]. Hence there is a need for an alternate anti-diabetic drug that works independent of the proximal insulin signaling and weight loss.

In animal models, human adenovirus Ad36 increases glucose uptake in cells [8, 10, 11] and improves glycemic control [12] while reducing hepatic lipid accumulation [13–15]. The 125 aa product of the E4orf1 gene of Ad36 is necessary and sufficient for virus-mediated cellular glucose uptake [9]. E4orf1 improves glycemic control in various mouse models [16–19]. In vitro, the E4orf1 peptide increases glucose uptake in the adipose and muscle tissues, and reduces glucose output from hepatocytes [8, 20–22]. E4orf1 does not seem to require the proximal insulin signaling for glucose uptake. In fact, E4orf1 inhibits IRS1 and IRS2 signaling by phosphorylating their serine residue [8]. Also, E4orf1 can increase cellular glucose uptake even when IR is knocked down by siRNA [8, 23]. Although IR was knocked down in this study [20], insulin-like growth factor 1 receptor (IGF-1R) likely remained functional, which could have contributed to cellular glucose uptake. IGF-1R is structurally similar to the insulin receptor (IR) [24] and insulin binds to both with varying affinity to initiate insulin signaling [2]. Inhibition of the IGF-1R causes insulin resistance with hyperglycemia [25] similar to when IR is impaired or inhibited [26]. Therefore, it was unknown if E4orf1-induced glucose uptake when IR was knocked down is completely independent of the proximal insulin signaling or used the IGF-1R signaling. It was also unclear if E4orf1 requires the distal insulin signaling pathway for enhancing glucose uptake.

This study separately determined the requirement of the proximal or distal insulin signaling for E4orf1 to increase cellular glucose uptake. First, we combined chemical inhibitors of IR and IGF1-R, (S961 and PPP, respectively), to block the proximal insulin signaling in 3T3-L1 cells, while leaving the distal insulin signaling intact. Separately, we used WM to block the distal insulin signaling, but left the proximal insulin signaling functioning. In presence of these various inhibitors of insulin signaling, we determined the effect of E4orf1 on glucose uptake and insulin signaling.

## Materials and methods

Experimental outlines are described below. Details of assays are presented under “Techniques and assays” (T&A) section.

Briefly, 3T3-L1 cells that express a null vector (NV) or Ad36E4orf1 (E4) induced in response to doxycycline exposure (stable cell lines developed as previously described- [27]) were treated with S961 or PPP or a combination of the two to completely block the proximal insulin signaling as described in T&A. Separately, the cells were exposed to WM to determine the role of distal insulin signaling in E4orf1 related glucose uptake. Cellular glucose uptake was determined in absence and presence of insulin. Protein lysates from E4 and NV cells were separated by SDS-PAGE and used for western blot analysis. Membranes were immunoblotted with either pAKT or pAS160 and normalized to total AKT or total AS160, respectively, to determine changes in protein expression as described in T & A. In a parallel experiment, treated E4 and NV cells were subjected to flow cytometry analysis to determine the translocation of Glut4 from the cytoplasm to the membrane.

### Techniques and assays

#### Cell culture

E4 and NV cells were maintained in Dulbeco’s Minimum Essential Medium (DMEM. Gibco #11995) supplemented with 10% Tetracyclin-free FBS (fetal bovine serum) (Clontech Laboratories, #631101), 1% Penicillin-Streptomycin (Sigma #P4333), Puromycin (Invivogen, # ant-pr-1) and Hygromycin (Thermofisher # 10687010). The two cell lines were seeded in 6 well plates and E4orf1 expression induced with 1000 ng/ml Doxycycline (Sigma # D3072-1ML) for 24 h. Cells were serum starved for 16 h during doxycycline induction, followed by treatment with inhibitors for insulin receptor S961(Phoenix Pharmaceuticals # 051–86), IGF-1 receptor- Picropodophyllin, (PPP) (Santacruz # sc-204008), or PI3K-Wortmannin (WM) (Selleckchem # S2758). Exposure to inhibitors was 2 h, except WM for 30 min and cells were exposed to 100 nM insulin for 20 min. One group each of E4 and NV cells were not exposed to insulin to determine baseline response.

To determine the dose and treatment duration for IR, IGF-1 and PI3K inhibitors, NV cells were starved for either 2, 16 h or not starved, in the presence or absence of doxycycline (1000 ng/ml). Based on literature [26, 28–30], different concentrations 100, 150 and 200 nM of S961 were tested for either 2 or 5 h. PPP and Wortmannin were tested for 30 min, 2 h and 5 h. pAkt /Akt analysis of all these conditions showed that 16 h of starvation in the presence of doxycycline (24 h induction) worked best for all the inhibitors. 200 nM S961 and 1 μM PPP for 2h and 100 μM Wortmannin for 30 min showed significant pAkt /Akt reduction (data not shown). All treatment groups were exposed to 100 nM insulin for 20 minutes. The conditions above were used to further test pAkt/Akt levels in E4 cells.

#### Glucose uptake assay

Treated and serum starved cells were washed twice with 1X PBS followed by addition of Krebs-Ringer phosphate (KRP) buffer (900 μL) with 0.2% BSA [9]. For insulin stimulation, 100 nM insulin (Sigma # I0516) was supplemented in KRP buffer and cells were incubated for 20 min. To determine non-specific glucose uptake, cells were treated with 100 nM cytochalasin B (Sigma Aldrich #6762). Following incubation for 20 min, 100 μL of 10X isotope solution was added to each well for a final concentration of 100 nM cold 2-deoxy glucose (Sigma # D6134) and 0.5 mCi/mL [3H]- 2-Deoxyglucose (PerkinElmer #NEC720A250UC) for 5 min. Cells were immediately washed in ice cold 1X PBS (3X) at the end of 5 min and plates air-dried. To lyse cells, 1 mL of 0.05% SDS was added to each well and incubated at 37°C for 45–60 min. Protein lysates (900 μL) were added to individual scintillation vials containing 3 mL of scintillation liquid (Wheaton #986540) and 50 μL of sample used for protein estimation by bicinchoninic acid (BCA) assay. Radiolabeled glucose in cell lysate was measured using a Beckman scintillation counter (Perkin Elmer TriCarb 4810TR) and scintillation counts per minute were normalized to protein content of each well.

#### Western blot

Treated cells were lysed in RIPA buffer (Enzo LifeSciences # ADI-80-1496) with protease inhibitors (Sigma # 5892791001) at 4°C for 30 min with shaking. Protein from the cell lysates was collected by centrifugation at 13000 rpm for 10 min at 4°C. Protein estimation of the cell lysates was performed by bicinchoninic acid (BCA) assay (Thermo Fisher Scientific # 23225) and 30 μg of protein lysates were separated by SDS-PAGE electrophoresis in a 12% gel. Separated proteins were transferred onto PVDF membrane and immunoblotted with either pAKT antibody (Cell Signaling # 9271L) 1:250 dilution or pAS160 (Thr642) (Cell Signaling #4288S) 1:250 dilution. The blot was stripped and immunoblotted with either total AKT (Cell Signaling #4691S) 1:1000 dilution or AS160 (Cell Signaling #2670S) 1:1000 dilution. The expression of pAKT was normalized to total AKT and pAS160 was normalized to total AS160.

#### Flow cytometry

Treated E4 and NV cells were trypsinized and blocked in blocking buffer (PBS/3% FCS) for 30 min at 4°C. Following blocking, cells were centrifuged at 5000 rpm, 4°C, for 5 min, supernatant was aspirated and cells were incubated with Glut4 antibody (1:50) (Abcam #ab65267) in PBS/3% FBS for 1 h at 4°C, and washed 3X with PBS/3%FBS. Cells were stained with anti-mouse Alexa 647 antibody (Thermofisher Scientific #A31571, 1:50) at 4°C for 1 h, washed 3X with PBS/3% FBS, and fixed with 2% paraformaldehyde for 15 min at RT. Cells were analyzed with flow cytometer (Attune NxT, Thermofisher Scientific) with cytometer settings for both forward scatters (FSC) and side scatters (SSC) set to 80 and 600 respectively to analyze cells. For each sample, 5000 cells were counted.

## Results

**Fig 1:**
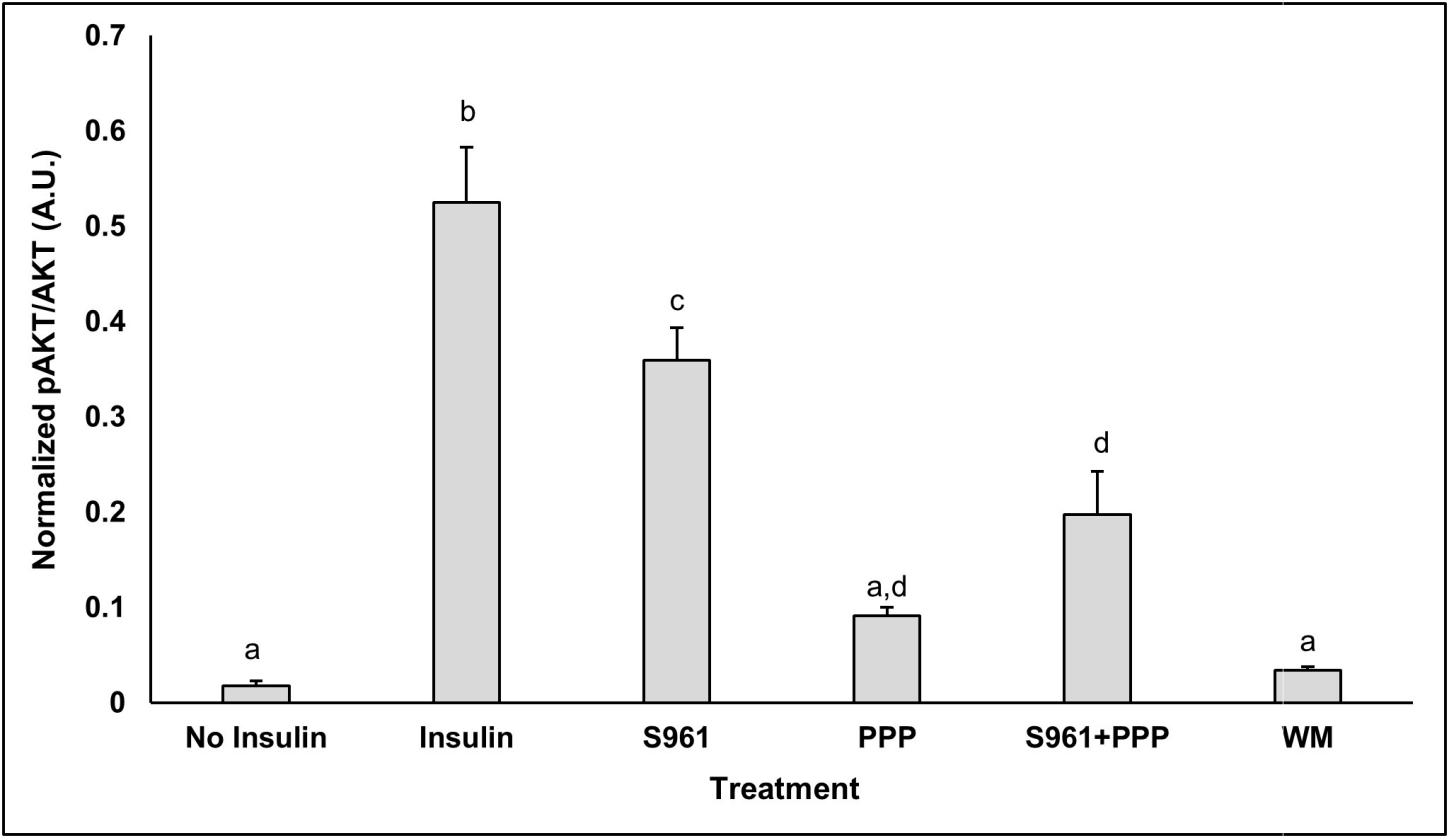
Potential of S961, PPP or WM to downregulate insulin signaling. pAKT abundance, normalized to AKT abundance was determined in NV cells treated with no insulin (Control), insulin or treated with insulin plus IR inhibitor S961, or IGF-1R inhibitor (PPP), S961+PPP combined (S961+PPP), or PI3K inhibitor wortmannin (WM). The treatments were compared using one-way ANOVA and post hoc Tukey’s HSD test, p≤0.05. Treatment sharing letters are not statistically significant.

The response of E4 and NV cells in terms of cellular glucose uptake, pAKT and pAS160 protein abundance and Glut4 translocation under intact or impaired insulin signaling was as follows:

**Potential of S961, PPP or WM to downregulate insulin signaling** (Fig 1)**:** As indicator of insulin signaling, pAKT abundance was determined in 3T3-L1 cells (NV) exposed to 0 or 100 nM insulin and no inhibitors, or in presence of S961, PPP, S961+PPP or WM. As expected, 100 nM insulin significantly increased pAKT abundance (Fig 1). Exposure of cells to each of the inhibitors successfully blocked insulin signaling, as indicated by failure of insulin to increase pAKT abundance (Fig 1).

**Fig 2:**
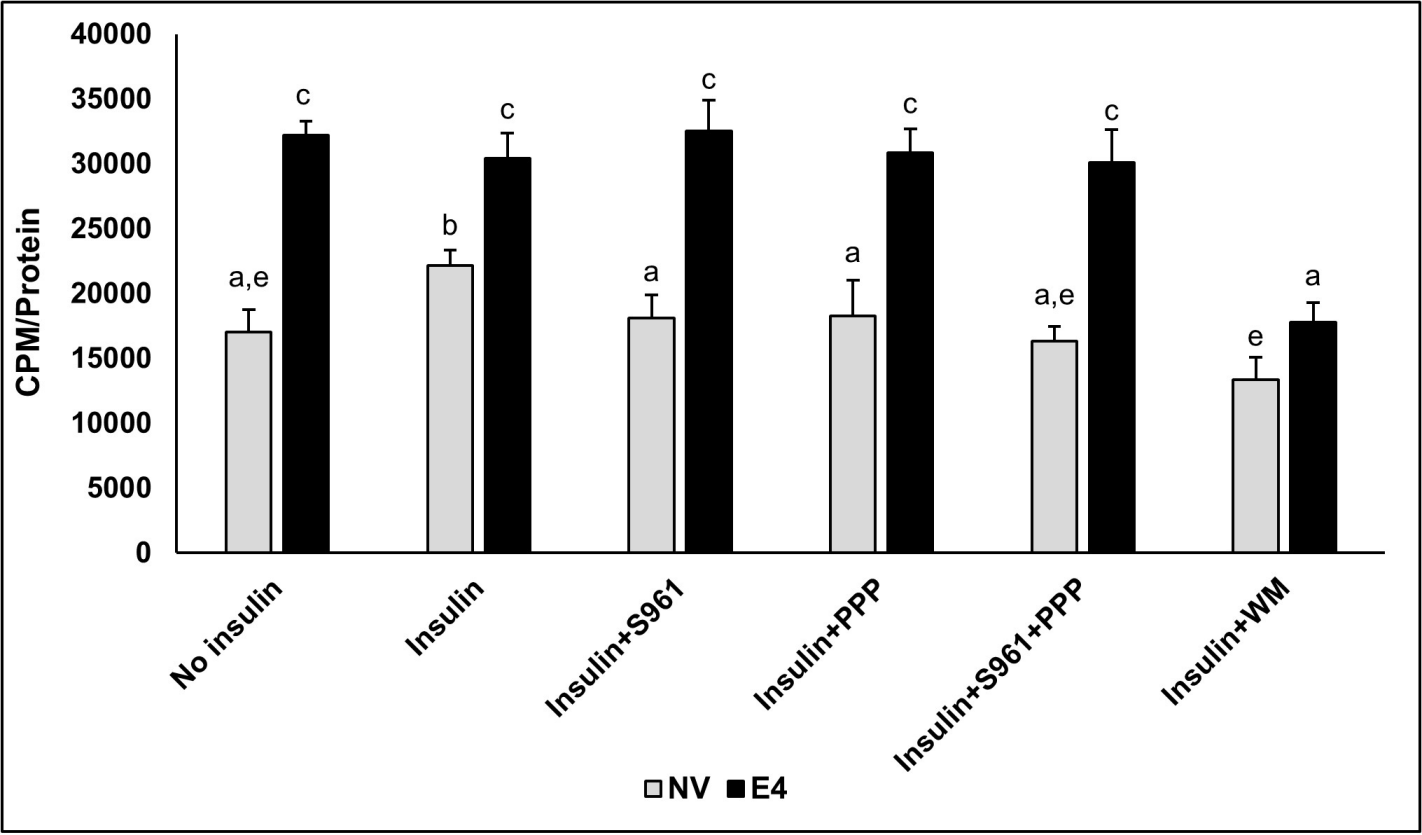
Glucose uptake in presence of the inhibitors of proximal or distal insulin signaling. Cellular glucose uptake was determined in NV and E4 cells treated with no insulin (Control), or treated with insulin plus IR inhibitor (S961), IGF-1R inhibitor (PPP), S961+PPP combined (S961+PPP), or PI3K inhibitor (WM). The treatments were compared using one-way ANOVA and post hoc Tukey’s HSD test, p≤0.05. Treatment sharing letters are not statistically significant.

**Glucose uptake with and without intact insulin signaling** (Fig 2)**:** As expected, in the absence of insulin signaling inhibitors, glucose uptake by E4 was significantly greater at baseline and under insulin stimulated conditions compared to NV cells in respective groups. In NV cells, exposure to insulin increased glucose uptake, only in the absence of insulin inhibitors. Exposure to S961, PPP, S961+PPP or WM abrogated insulin-stimulated glucose uptake in NV cells. Whereas, S961 or PPP exposure, alone or combined, did not decrease glucose uptake in E4 cells, compared to NV cells. WM attenuated glucose uptake in E4 cells, compared to the baseline glucose uptake in E4 cells.

**Fig 3:**
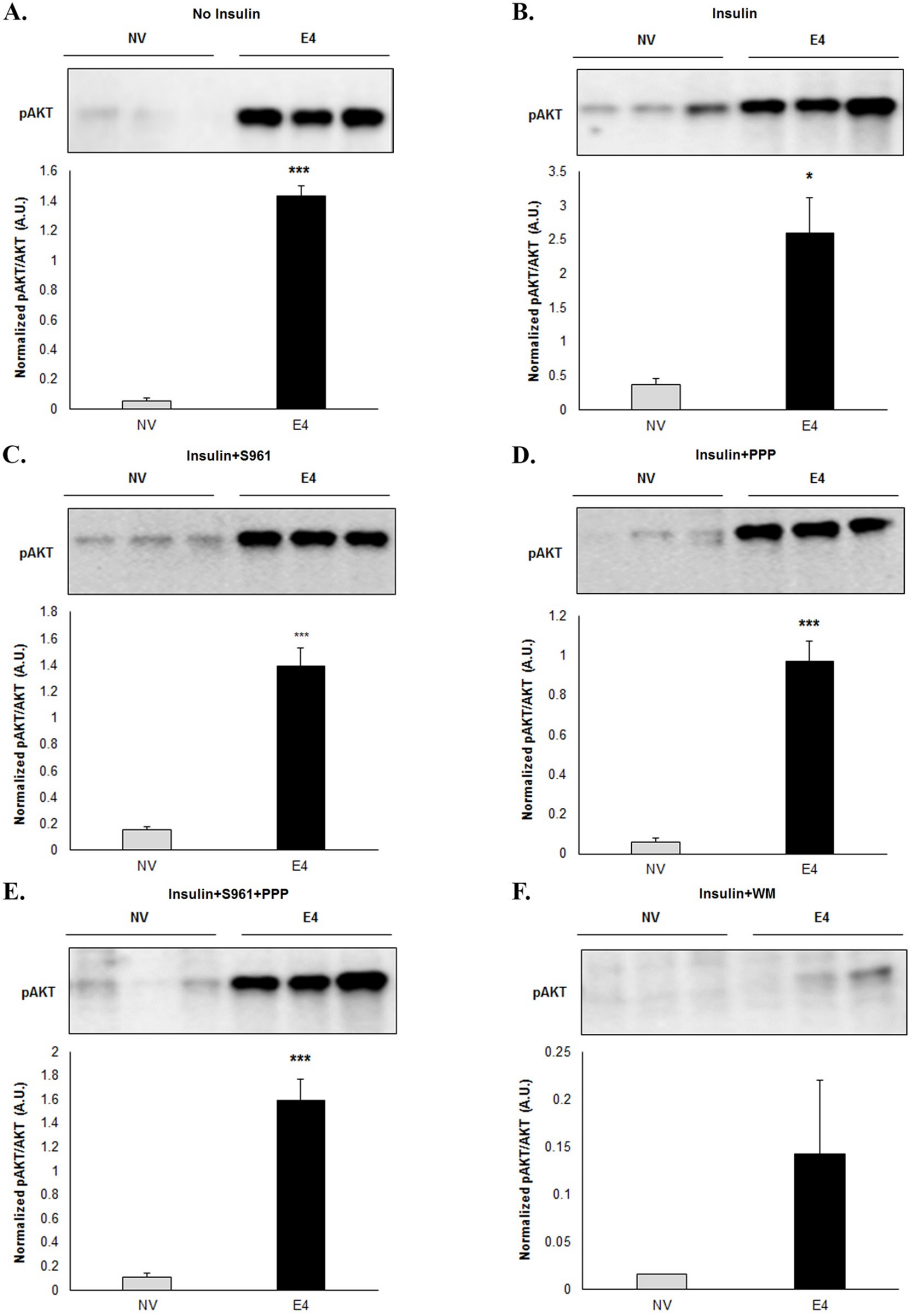
pAKT abundance in presence of the inhibitors of proximal or distal insulin signaling. pAKT abundance was determined in NV and E4 cells treated with no insulin (Control, A), insulin (B) or treated with insulin plus IR inhibitor (S961, C), IGF-1R inhibitor (PPP, D), S961+PPP combined (S961+PPP, E), or PI3K inhibitor (WM, F). The cell lysates were separated by SDS-PAGE and immunoblotted with pAKT and total AKT antibody. The E4 cells were compared to NV cells using t-test, * p≤0.05, *** p≤0.001.

**pAKT abundance with and without intact insulin signaling** (Fig 3): In the absence of insulin signaling inhibitors, pAKT abundance in E4 cells was significantly greater with or without insulin, compared to NV cells in respective groups. Compared to NV cells, E4 cells continued to show significantly greater pAKT abundance even after exposure to S961, PPP, or S961+PPP, but not upon WM exposure.

**Fig 4:**
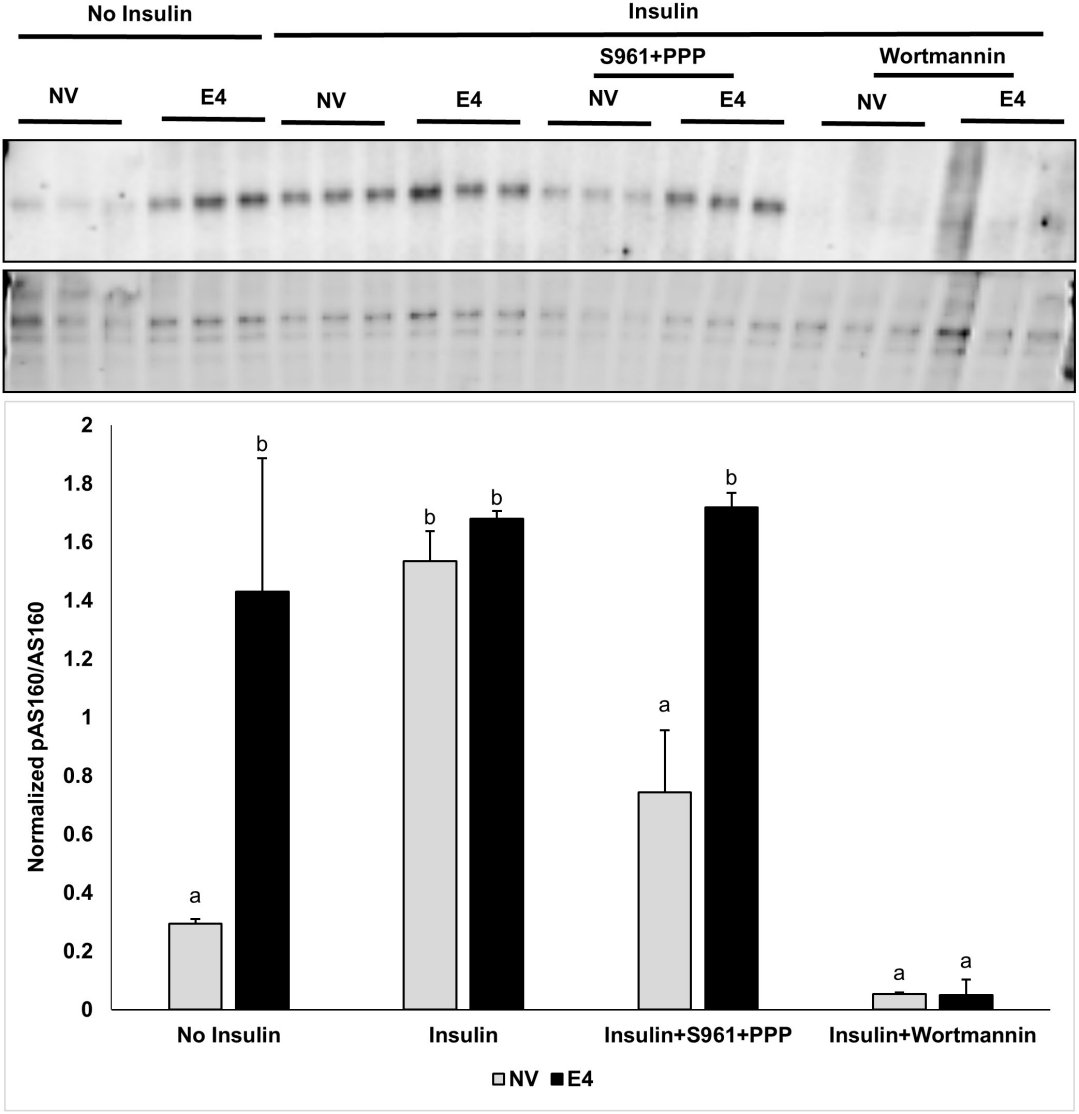
pAS160 abundance in presence of the inhibitors of proximal or distal insulin signaling. pAS160 abundance was determined in NV and E4 cells treated with no insulin or with insulin or insulin plus IR inhibitor (S961) and IGF-1R inhibitor (PPP) combined or PI3K inhibitor (WM). The cell lysates were separated by SDS-PAGE and immunoblotted with pAS160 and total AS160 antibody. The E4 cells were compared to NV cells using one-way ANOVA and post hoc Tukey’s HSD test, p≤0.05. Treatment sharing letters are not statistically significant.

**pAS160 levels with and without functional insulin signaling** (Fig 4)**:** During cellular glucose uptake, phosphorylation of AKT leads to phosphorylation of AS160, activating translocation of Glut4 to the membrane for uptake of glucose [31, 32].

Here, we observed that as expected, compared to no-insulin baseline, insulin increased pAS160 abundance, but not if insulin signaling was blocked by S960 and PPP or WM. pAS160 abundance in E4 cells was significantly greater with or without insulin, compared to NV cells in respective groups. Compared to NV cells, E4 cells continued to show significantly greater pAS160 abundance even after exposure to S961+PPP, but not upon WM exposure.

**Fig 5:**
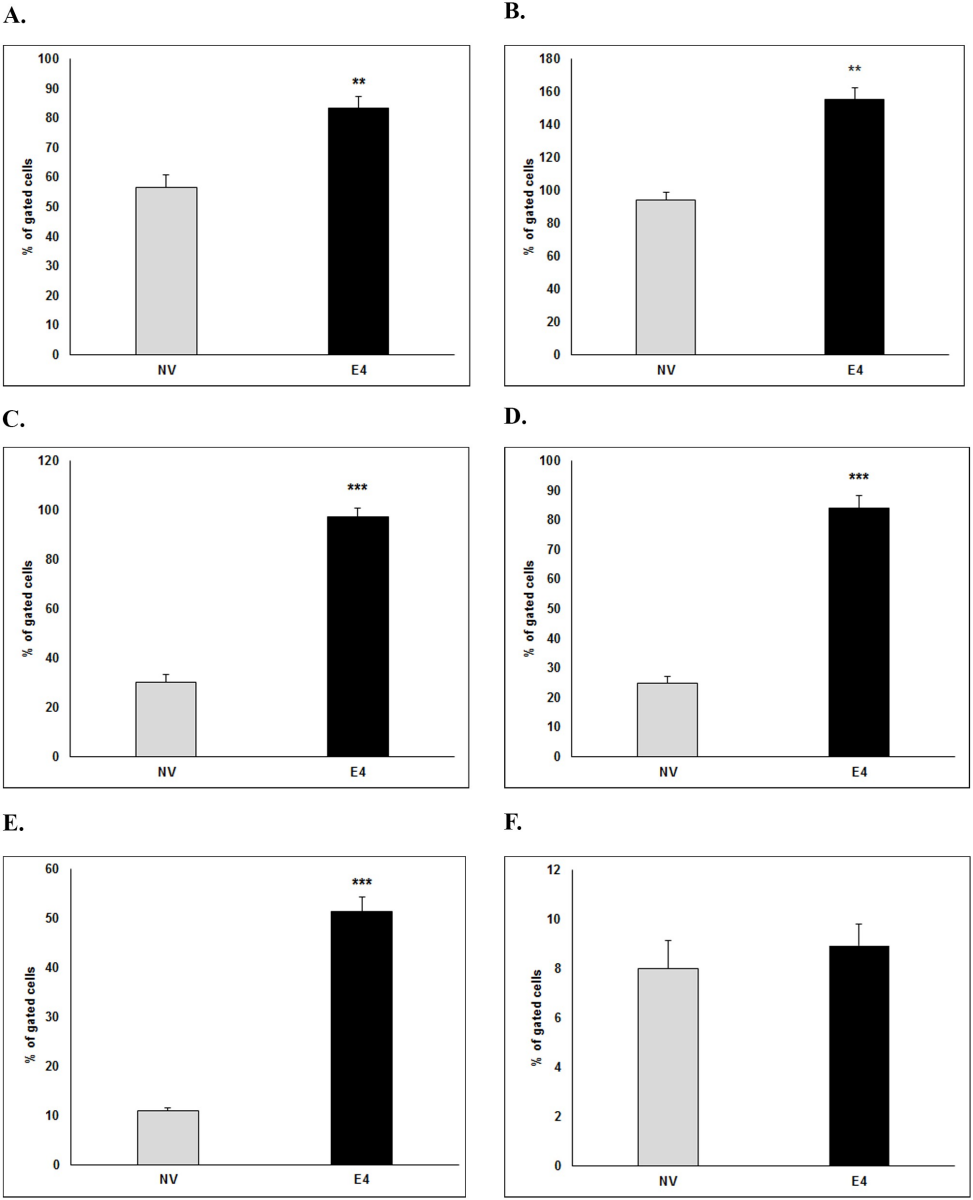
Glut4 translocation in presence of the inhibitors of proximal or distal insulin signaling. Translocation of Glut4 to cell membrane was determined in NV and E4 cells treated with no insulin (Control, A), insulin (B), or treated with insulin plus IR inhibitor (S961, C), IGF-1R inhibitor (PPP, D), S961+PPP combined (S961+PPP, E), or PI3K inhibitor (WM, F). Cells were trypsinized and stained with Glut4 antibody, followed by Alexa Fluor 647 secondary antibody. Stained cells were analyzed with flow cytometry to detect Glut4 on cell membrane. The E4 cells were compared to NV cells using t-test, * p≤0.05, *** p≤0.001.

**Glut4 translocation with and without intact insulin signaling** (Fig 5): In the absence of insulin signaling inhibitors, Glut4 translocation to the cell membrane in E4 cells was significantly greater with or without insulin, compared to NV cells in respective groups. Compared to NV cells, E4 cells continued to show significantly greater Glut4 translocation even after exposure to S961, PPP, or S961+PPP, but not upon WM exposure.

## Discussion

“*Despite the abundance of FDA-approved therapeutic options for type 2 diabetes*, *the majority of American patients with diabetes are not achieving appropriate glycemic control*. *The development of new options with new mechanisms of action may potentially help improve outcomes and reduce the clinical and cost burden of this condition*” [33, 34], -this is an often expressed need in scientific literature. We are working on E4orf1 protein, a new candidate as a potential anti-diabetic agent.

Studies with Ad36 natural infection in humans and experimental infection of animals (chickens, rats, mice, non-human primates) showed that inspite of correlatively and causatively linked with obesity (review- [35, 36], Ad36 infection improved glycemic control and attenuated hepatic steatosis in rodents [12, 13]. Subsequent in vitro studies, identified *E4orf1* gene of Ad36 as necessary and sufficient for the effect of the virus on glucose disposal [21, 22, 27, 37].

Recent reports in mice models have strongly highlighted the role of E4orf1 peptide of Ad36 as a very promising therapeutic candidate for Type 1 or Type 2 diabetes [16–19]. E4orf1 expression in these mice significantly reduces glucose excursion compared to control [17], yet lowers the insulin response to glucose load, indicating a reduced need for insulin. In theory, the reduced endogenous insulin in response to E4orf1 could either be due to increased tissue sensitivity to a given amount of insulin, reduced insulin production or secretion, or due to the ability of E4orf1 to enhance cellular glucose uptake independent of insulin. Of these possibilities, the increased tissue sensitivity to insulin due to E4orf1 is unlikely as E4orf1 downregulates proximal insulin signaling [23, 38]. Also, the reduced insulin secretion in response to exogenous glucose is not due to pancreatic beta cell damage [19]. This collectively indicates a reduced need for insulin in presence of E4orf1, termed as the “insulin sparing effect” of E4orf1 [38]. These properties make E4orf1 a valuable candidate to investigate for its anti-diabetic effects.

The down-regulation of proximal insulin signaling by E4orf1 [23], coupled with reduced insulin release and yet enhanced glucose disposal suggested that at least a portion of insulin signaling pathway is not needed for E4orf1 induced glucose disposal. As a potential anti-diabetic agent, this property of E4orf1 may be highly attractive for a couple of reasons. While the impairment in insulin signaling pathway that contributes to insulin resistance may vary between individuals, broadly speaking, impairments in proximal [2, 3], or distal insulin signaling have been recognized [39, 40]. This underscores the need to develop anti-diabetic agents that would improve glycemic control without recruiting the proximal or distal insulin signaling, which may be impaired. Hence, it would be important to understand the part of the insulin signaling that E4orf1 bypasses or engages in improving glucose disposal.

The molecular events that lead to E4orf1-induced glucose uptake can be surmised as follows [18, 21, 22, 41–44]: In its interaction with insulin signaling pathway, E4orf1 inhibits the proximal insulin signaling by reducing tyr-phosphorylation of IRS, and by increasing its ser-phosphorylation. E4orf1 enters insulin signaling at the level of PI3K activation. E4orf1 has a PDZ domain binding motif (PBM) at the C-terminal. Through its PBM, the peptide binds to PDZ proteins, which are scaffolding proteins that facilitate protein–protein interactions. E4orf1 requires its PBM to bind to Dlg (Drosophila disc large)-1 protein. After the binding, the complex travels to the cell membrane, where it activates Ras and subsequently, up-regulates PI3K-AKT signaling, leading to greater membrane translocation of Glut4, and glucose uptake.

**Fig 6:**
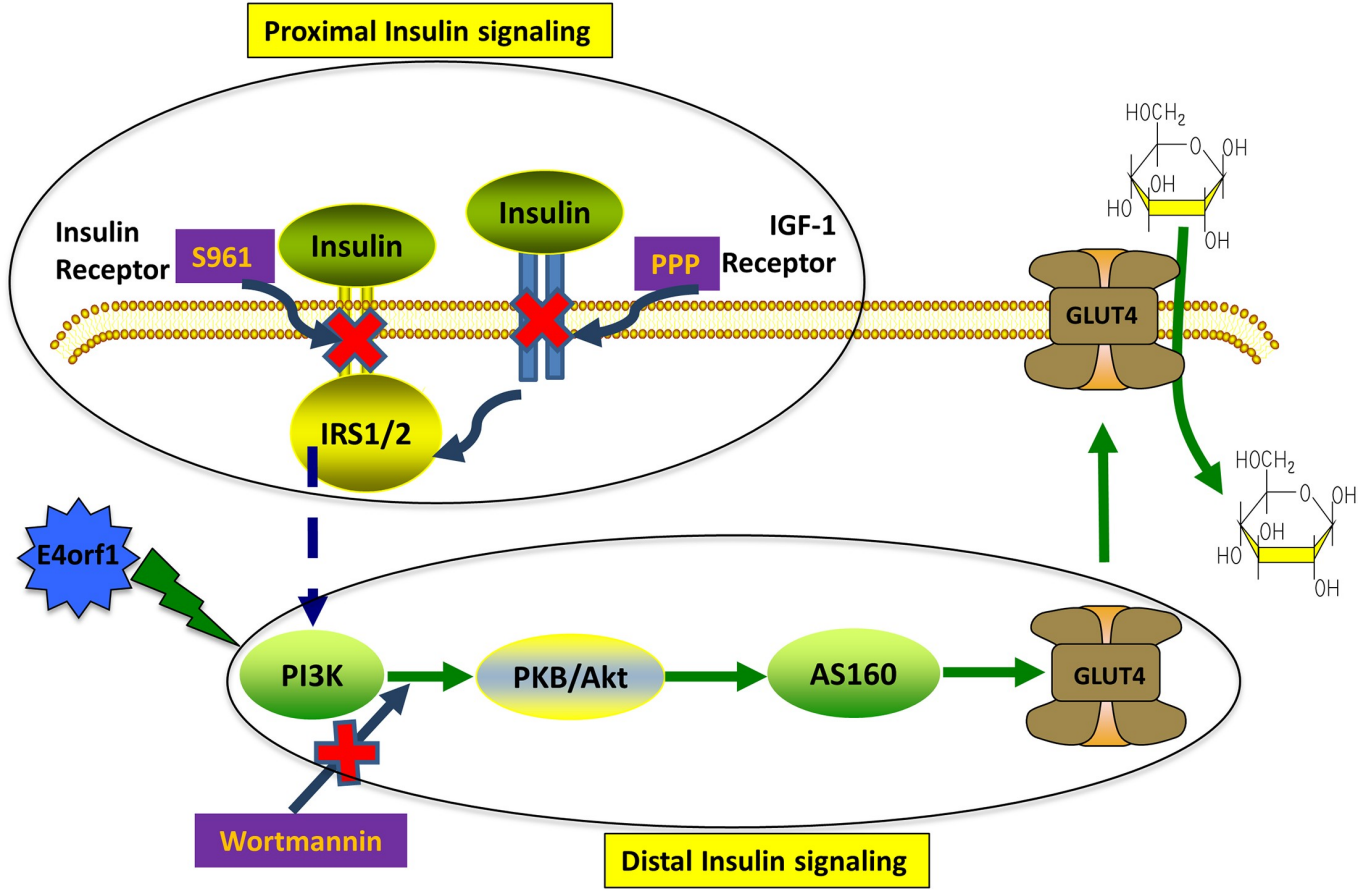
E4orf1 activates the distal insulin signaling for cellular glucose uptake when the proximal insulin signaling is impaired or blocked. This schematic shows E4orf1 specifically activates the distal insulin signaling by increasing AKT phosphorylation, AS160 phosphorylation and Glut4 translocation for glucose uptake under conditions when proximal insulin signaling pathway is inhibited with insulin receptor (S961) and IGF-1 (PPP) agonist. However E4orf1 is unable to overcome a block to the distal insulin signaling by WM, highlighting it specific role in activating this pathway.

Based on this information, we hypothesized that E4orf1 bypasses the proximal insulin signaling, but requires the distal insulin signaling pathway for enhancing cellular glucose uptake. Here, we investigated glucose uptake, AKT and AS160 activation and Glut4 translocation in 3T3-L1 cells when the proximal insulin signaling was inhibited by IR and IGF-1R antagonists or the distal insulin signaling was inhibited by WM. Our results showed that as expected, in 3T3-L1 cells the presence of E4orf1 increased AKT phosphorylation, increased AS160 phosphorylation, Glut4 translocation and glucose uptake in basal or insulin stimulated conditions or even after inhibiting the proximal insulin signaling with S961 and PPP. However, E4orf1 could not increase glucose uptake or Glut4 translocation, when the distal insulin signaling was inhibited by WM (Fig 6). While this study investigated the role of 3T3-L1 preadipocytes, it would be useful to determine if the skeletal muscle cells and liver cells respond similarly. The applicability of the findings to humans is unknown. The next step would be to test if an animal model replicates the findings.

Collectively, the results indicated that potentially, E4orf1-based therapeutic agent may be especially beneficial for individuals with impaired proximal insulin signaling, but intact distal insulin signaling pathway. E4orf1’s insulin sparing action together with its ability to bypass the proximal insulin signaling and improve cellular glucose uptake, makes it a potential candidate for the development of an anti-diabetic drug.
